# Recurrent Echinococcosis Exhibiting Hydatoptysis: A Rare Case with Imaging Insights

**DOI:** 10.3390/jpm14080796

**Published:** 2024-07-27

**Authors:** Maria Konstantinidou, Eleni D. Eleftheriadou, Effimia Kamariotou, Christina Rampiadou, Styliani Papaemmanouil, Diamantis Chloros

**Affiliations:** 1 Department of Respiratory Medicine, G. Papanikolaou General Hospital, 57010 Thessaloniki, Greece; heleneleft@gmail.com (E.D.E.); efi_kamariwtou@hotmail.com (E.K.); ram.christina89@gmail.com (C.R.); dchloros@msn.com (D.C.); 2 Department of Pathology, G. Papanikolaou General Hospital, 57010 Thessaloniki, Greece; lianapapaem@yahoo.gr

**Keywords:** echinococcosis, tropical disease, hydatidosis, hydatoptysis, bronchial fistulization, hookworms, chitin layers

## Abstract

Echinococcosis presents diverse clinical manifestations, including pulmonary hydatidosis, often asymptomatic but complicated by rare yet dramatic occurrences like hydatoptysis. Here, we report a unique case of recurrent pulmonary and abdominal hydatidosis in a 59-year-old female with bronchial fistulization and hydatoptysis, despite previous surgical interventions. Imaging revealed characteristic features aiding in diagnosis and management decisions. The challenges in managing recurrent echinococcosis underscore the importance of comprehensive follow-up and multidisciplinary care. Surgical intervention remains pivotal, supplemented by medical therapy with careful consideration of risks and benefits. This case also presents rare images, absent in much of the literature, which provide valuable insights into the disease’s presentation and progression.

## 1. Introduction

Echinococcosis is a parasitic tropical disease, with Echinococcus granulosus and less commonly Echinococcus multilocularis being the usual responsible species, with different geographical distribution and clinical manifestations. The cycle of life of echinococcus is well described in the literature [1,2]. Pulmonary hydatidosis is usually asymptomatic and randomly diagnosed; however, a noisier clinical image emerges when the disease is complicated, with rupture being the commonest complication [3]. Hydatoptysis is a rare manifestation of a complete rapture into a bronchiole. It is characterized by expectoration of germinative membranes, hooklets or daughter cysts with cough, leaving a salty or bitter taste in the mouth [4,5]. Diagnosis is managed with a combination of clinical history, imaging features and serological findings [6]. Surgery in combination with anthelmintics is the treatment of choice even in uncomplicated cysts [7]. Although it is a nonmalignant disease, the best care is to perform radical and curative excisions; otherwise, the disease is associated with a high recurrence rate [8]. Herein, we present an unusual case of recurrent synchronous pulmonary and liver hydatid cysts, accounting for less than 10% of cases reported in the literature [9]. This case was further complicated by cyst rupture into the bronchiole, leading to bronchial fistulization and hydatoptysis.

## 2. Case Presentation

**Figure 1:**
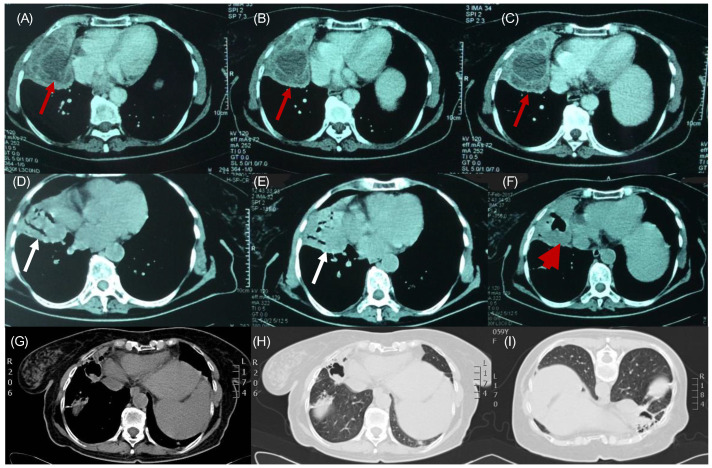
*1 year prior:* (**A**–**C**): Multiple unraptured hydatid cysts, containing daughter cysts. The fluid inside the cysts has a water density (red arrows). During hospital stay: (**D**,**E**): Combo or onion peel sign. We see air–fluid levels in the endocyst and pericyst (white arrows). (**F**): Water-lily sign or camalote sign. Complete rapture of the endocyst. Membranes float in the remaining fluid. The last two signs indicate complete rapture (red arrowhead). Follow-up: (**G**) Thorax CT 6 months after treatment: Intracancerated membrane sign or mass in the cavity sign on the mediastinal window, (**H**) on the lung-on-lung window, and (**I**) at prone position, the remaining solid components wrinkle to the dependent wall of the cyst.

**Figure 2:**
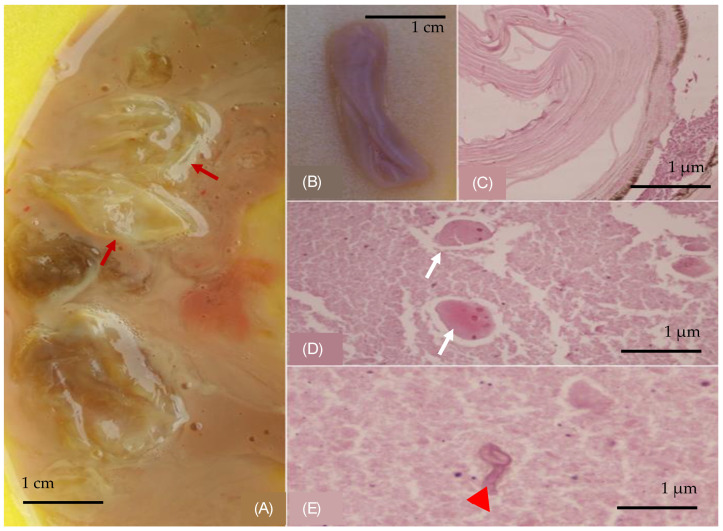
(**A**) Membranes in expectorated content (red arrows). (**B**) A collapsed grayish and lilac-colored gelatinous membrane, part of a cystic wall, with a median diameter of 3 cm. Histological examination: (**C**) wall of a cystic-shaped morphoma composed of multiple chitin layers, (**D**) eggs (white arrows) and (**E**) hookworms (red arrowhead). (Hematoxylin and Eosin stain).

A 59-year-old female patient who had a protracted history of echinococcosis, presenting with fever and intermittent cough. She had a residence history in a rural community in Northern Greece, with close contact with dogs during her upbringing. Her medical history documented multiple rounds of anthelmintic therapy and nine surgeries, performed within the last 15 years addressing cystic echinococcosis, primarily affecting the right lung, liver and abdomen, albeit with limited success. One year prior to the admission, she was hospitalized to a regional hospital due to disseminated echinococcosis, with a positive serum test for Immunoglobulin-G enzyme-linked immunosorbent assay (ELISA) for Echinococcus. A computed tomographic (CT) scan of the thorax, conducted during that admission, revealed the presence of multiple cysts with thin-walled calcified rims, localized in the anatomical region of the middle lobe. These cysts exhibited characteristic water attenuation with no discernible septations (Figure 1A–C). The lesions infiltrated the diaphragm, the liver, and multiple intra-abdominal hydatic cysts were observed. She received an extended course of albendazole consisting of 3 treatment cycles at a dose of 400 mg taken orally twice daily. During her present admission to our tertiary hospital, due to lower respiratory infection, the patient presented with a 10-day history of fever and intermittent cough, despite undergoing a treatment regimen that included oseltamivir and azithromycin, followed by moxifloxacin, yielding no clinical improvement. Upon examination, she displayed full orientation, hemodynamic stability, with a recorded temperature of 38.5 °C, devoid of chills or diaphoresis. The respiratory rate was 16 breaths per minute, and the oxygen saturation 98% on ambient air. The pulmonary auscultation revealed dullness at the midpoint of the right lung accompanied by normal breath sounds. Laboratory investigations unveiled an absolute eosinophilic count of 480/μL and Erythrocyte Sedimentation Rate of 140 mm/h. All the other hematological and biochemical parameters were within the normal range. Her chest radiography disclosed air space consolidation with air-bronchogram in the middle lobe in addition to blunting of both costophrenic angles. Unexpectedly, on the second hospital day, subsequent to a brief episode of intense cough, she developed hydatoptysis, expelling a substantial amount of watery fluid containing rounded membranes and droplets of blood (Figure 2A,B). Microscopic examination of the specimens revealed remnants of hooklets in the sputum, while histology showed pieces of cystic layer comprising of chitin petals and protoscoleces (Figure 2C–E). The Gram stain was negative despite detecting numerous pyospheres as well as staphylococcus and streptococcus. Subsequently, the patient underwent a repeat CT scan that identified a large, complicated cyst displaying an air–fluid level within it. Importantly, the cyst exhibited communication with the bronchi indicative of bronchial fistulization (Figure 1D–F). Furthermore, the CT scan unveiled multiple homogeneous-shaped water-dense cysts in the liver, characterized by perfectly defined margins, and the presence of air bubbles. Based on these findings, the patient received a definite diagnosis of infected disseminated, recurrent pulmonary and abdominal hydatidosis. Consequently, she was promptly referred to surgical consultation for further evaluation and management; however, she refused further surgical treatment. Therefore, she received continuous therapy with albendazole for 6-month cycles at a dose of 400 mg taken orally twice daily, to address the parasitic infection. On follow-up, she was asymptomatic, without exhibiting a new episode of hydatoptysis, and there was an improvement in CT imaging with signs of complete rapture and drainage of the cysts. The greatest cyst had been shrunken, and the membranes were wrinkled and fell to her dependent wall, creating a mass in the cavity or intracancerated membrane sign (Figure 1G–I).

## 3. Discussion

The peculiarity of this recurrent echinococcosis case is the availability of images from the sputum sample and its analysis. Despite extensive prior interventions, our patient suffered recurrence of pulmonary and abdominal hydatidosis, characterized by bronchial fistulization and reaching its apex in the dramatic manifestation of hydatoptysis. Pulmonary cystic echinococcosis is mainly a well-studied tropical disease that has occupied the medical field for many years [3]. There is no standard for the management of cystic echinococcosis of the lung [10]. Hence, even though surgery is considered the treatment of choice, the timing of intervention, the type of surgery, and the need for additional treatment are often subjected to the experience of the respective center [11]. Although it is a nonmalignant disease, the best care is to perform radical and curative excisions; otherwise, the disease is associated with a high recurrence rate [8]. In large studies, the recurrence rate was approximately 3–5% [12,13,14]. Difficult-to-reach location of the cysts, intraoperative spillage of the cyst’s material, and the inability of the patients to undergo such procedures due to poor clinical condition are some of the conditions resulting in recurrence [15]. The mean time of recurrence ranges between 2 and 10 years [8]. Our patient experienced intervals of approximately 1 to 2 years between each recurrence episode. The chronicity of the disease requires a long-term follow-up, which is often difficult to achieve, as the majority of these patients live in rural areas, and echinococcosis requires treatment in specialized centers. Usually, the individuals are asymptomatic or display non-specific symptoms like cough, chest pain when the cyst is large or dyspnea, and there are no sensitive or specific markers to help optimally monitor the disease [3]. Even the antibody titles, which are a helpful diagnostic tool, are not associated with disease activity as they remain positive for years after the removal [8]. This observation implies that, for the diagnosis of relapse, only imaging methods can be employed. However, since most cases involve heavily treated patients, like the present case, the approach is individualized [15]. A noisier clinical image with fever, hemoptysis, or productive sputum emerges when the disease is complicated by various insults [3]. The most frequent complication is the cyst’s rupture, which occurs in about half of the patients, and there are three different types: contained, communicated, or direct rupture [4]. A contained rupture is less severe, as only the endocyst is affected and the pericyst remains intact. Direct rupture occurs when both the endocyst and the pericyst are tearing apart, resulting in spillage of the cyst’s content. This complication is more common in peripheral lesions due to deficient pericyst and little host tissue support. It can also be caused intraoperatively, causing life-threatening anaphylactic shock in addition to disease dissemination and recurrence [4]. Our patient was complicated with communicating intrapulmonary rupture, which consists of abruption of the endocyst and perforation of the cyst’s material via bronchial radicals that are incorporated in the pericyst. Rarely, it can be manifested with hydatoptysis, as seen in our patient [5]. The presence of hooklets or scolices in smears of the specimen during pathological analysis is specific to the diagnosis [16]. Such cases often require radical surgical intervention because remnants of the collapsed parasitic membranes retained in the lung could be the source of a secondary infection [5]. Chest CT is the preferable imaging technique in order to diagnose rupture, especially intrapulmonary. There are numerous pathological patterns such us the “meniscus sign”, “combo sign”, “serpent sign”, “water-lily sign” or “intracancerated membrane sign”, depending on the degree of rupture and air penetration [4]. In our case, CT scan revealed the “combo”, “water-lily” and “intracancerated membrane” signs. As far as treatment goes, the combination of surgery with anthelmintics is the treatment of choice even in uncomplicated cysts. Avoiding invasive management when cysts are uncomplicated leads to more postoperative complications when these cysts become complexed [7]. Recurrence often requires reoperations, which increase the mortality rate (6% for the second operation and 20% for the third operation). Medical treatment with anthelmintics serves as a crucial tool against pulmonary echinococcus in diverse stages, both as a prophylactic and definitive therapy [3,10]. In selected patients, with uncomplicated, small cysts and contraindications for surgery, continuous treatment with mebendazole or albendazole for at least 3 to 6 months is suggested. Cyclic therapy is no longer suggested due to less efficiency and no difference in adverse events in comparison to continuous therapy. Praziquantel is used in combination with the above treatment to enhance treatment effectiveness [10]. However, medical treatment is frequently associated with the risk of cyst rupture into the airway, probably due to an alteration in the structure of cyst walls along with an increase in intracystic pressure, which makes the cuticular membrane more vulnerable to degenerative changes [17]. It is usually seen 2–4 weeks after treatment initiation; however, there are also cases of later onset [18]. There is a lack of clarity concerning our patient’s adherence to prescribed pharmacological treatment and the extent of comprehensive follow-up.

## 4. Conclusions

In conclusion, while cystic echinococcosis is a rare but still present disease in the Mediterranean region, treatment protocols and clinical guidelines are not clearly established. This has a significant impact on patients’ quality of life and is associated with elevated morbidity and mortality rates, along with an increased incidence of relapse. It is important that echinococcus incidents be documented within a comprehensive database, accompanied by meticulous follow-up, in specialized centers with a multidisciplinary team, in order to manage these complex cases effectively.

## Data Availability

The data and materials used in this case report were obtained from the patient’s medical file and are available from the corresponding author upon reasonable request.
